# 559. A Nationwide Survey of COVID-19 Management in the Dominican Republic Over the Course of the Pandemic

**DOI:** 10.1093/ofid/ofab466.757

**Published:** 2021-12-04

**Authors:** Alfredo J Mena Lora, Rita Alexandra Rojas-Fermin, David de Luna, Stephanie L Echeverria, Yori Roque, Ruben Calcano, Claudia Blanco, Clevy Perez, Anny Hernandez, Adames Espinal, Carolina Coronado, Michelle Castro, Arelis Batista, Scott Borgetti, Susan C Bleasdale

**Affiliations:** 1 University of Illinois at Chicago, Chicago, IL; 2 Hospital General Plaza de la Salud, Santo Domingo, Dominican Republic; 3 Internist-infectologist, Santiago de los cabelleros, Santiago, Dominican Republic; 4 Saint Anthony Hospital, Chicago, IL; 5 Pontificia Universidad Catolica Madre y Maestra (PUCMM), Santiago, Santiago, Dominican Republic; 6 SDI, Santo Domingo, Distrito Nacional, Dominican Republic

## Abstract

**Background:**

COVID-19 was declared a global Public Health Emergency by the WHO in January 2020. Limited treatment options existed early in the pandemic. As COVID-19 spread across the globe and new therapeutics emerged, different interpretations of the literature grew, and major societies relayed conflictive recommendations. There is a paucity of data on COVID-19 management in low- and middle-income countries. As a result, we performed a nationwide survey of local treatment practices in the Dominican Republic (DR).

**Methods:**

We performed an anonymous survey of infectious diseases specialists in the DR and US. The survey collected hospital characteristics and COVID-19 management protocols during different quarters of 2020-21. Management was categorized by drug and disease severity based on supplemental oxygen requirements. A convenience sample in the US representing community and academic sites was surveyed for point comparison between the US and DR.

**Results:**

The survey was completed by physicians from a total of 11 sites located in 4 cities of the DR: Santo Domingo (3), Santiago (4), La Vega (2) and San Francisco (2). These cities were representative of all regions in the country. The survey included 7 (64%) hospitals with < 200 beds, 3 (27%) with 201-300 beds, and 1 (9%) with >400 beds. Seven (47%) were private, 2 (13%) public, and 6 (40%) were teaching hospitals. In the US, 2 academic hospitals with >400 beds and 2 community hospitals with < 200 beds in a major city were surveyed. Management of COVID-19 at sites in the DR and US throughout the pandemic is plotted in Figure 1. Remdesivir use by disease severity is plotted in Figure 2.

Figure 1. Management of COVID-19 at sites in the US and DR throughout the COVID-19 pandemic

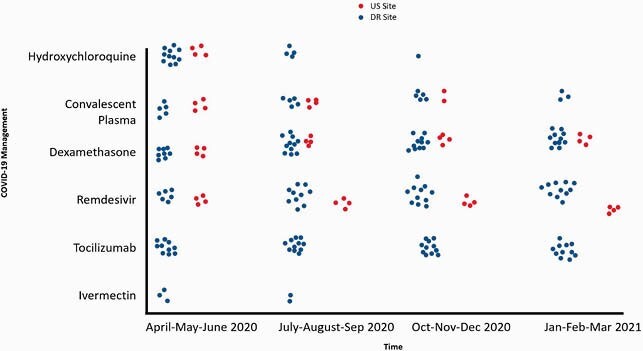

FIgure 2. Remdesivir use by disease severity at sites in the US and DR throughout the COVID-19 pandemic

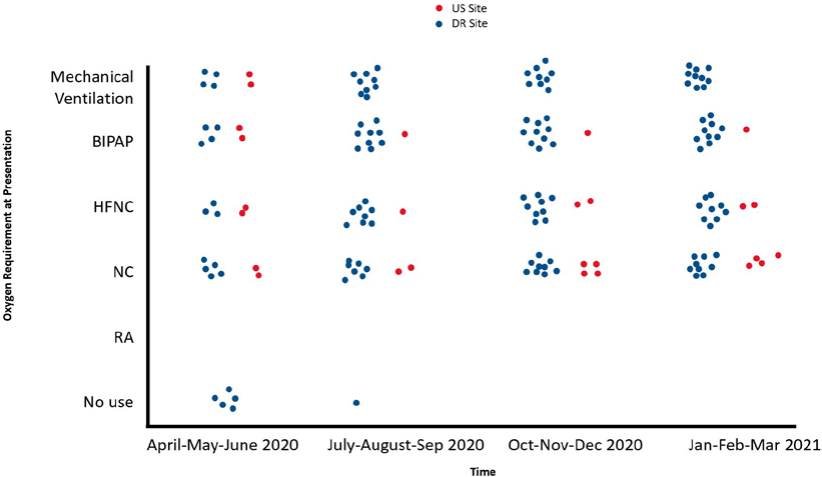

**Conclusion:**

Throughout the pandemic, as therapeutic options evolved, hospitals and physicians had to adapt to changing guidelines and availability of novel drugs. Variability between countries and sites emerged. The use of hydroxychloroquine and convalescent plasma waned more rapidly in the US. Dexamethasone was widely used at all sites. Tocilizumab and remdesivir were used more liberally in the DR. Antimicrobial stewardship limited these agents at US sites to more narrow therapeutic windows which could explain the discrepancies seen between the US and DR. Uncertainty of benefit in certain disease states, limited availability, and cost may also play a role.

**Disclosures:**

**All Authors**: No reported disclosures

